# Mesocolic schwannoma mimicking gastrointestinal stromal tumor: A case report and review of literature

**DOI:** 10.1097/MD.0000000000040434

**Published:** 2024-11-08

**Authors:** Qihang Sun, Qingshun Zhu, Xuren Lu, Guangxu Zhu, Wei Lang, Jie Zhang, Jianjun Qu

**Affiliations:** a General Surgery Department, Weifang People’s Hospital, Shandong Second Medical University, Weifang, Shandong, China; b Gastrointestinal Surgery Medical Center, Weifang People’s Hospital, Shandong Second Medical University, Weifang, Shandong, China; c Hypertension and Nephrology Department, Jinan Traditional Chinese Medicine Hospital, Jinan, Shandong, China.

**Keywords:** mesocolon, S-100, schwannoma, surgery

## Abstract

**Rationale:**

Schwannomas are common peripheral nerve tumors originating from Schwann cells, primarily occurring in the head and neck, limbs, and trunk. Schwannomas occurring in the mesocolon are rare and often have no specific manifestations. Abdominal schwannomas need to be differentiated from common abdominal tumors such as gastrointestinal stromal tumors.

**Patient concerns:**

We report a case of a mesocolic schwannoma in a 59-year-old female presenting with gastrointestinal symptoms of acid reflux. At an outside hospital, gastroscopy, colonoscopy, and abdominal computed tomography scans revealed a soft tissue mass adjacent to the greater curvature of the stomach, leading to a suspicion of a gastric mesenchymal tumor.

**Diagnoses:**

Mesocolic schwannoma.

**Interventions:**

Laparoscopy was performed at our hospital. Intraoperatively, the tumor was found to be closely related to the transverse colon and was initially diagnosed as a mass originating from the transverse colon. Consequently, a resection of the mass along with the adherent portion of the transverse colon was performed. Postoperative pathology and immunohistochemistry confirmed that the tumor was a schwannoma of the mesentery and did not originate from the transverse colon.

**Outcomes and lessons:**

Schwannomas can be distinguished from gastrointestinal stromal tumors by immunohistochemical staining, and surgical treatment is effective for benign schwannomas.

## 1. Introduction

Schwannomas are common peripheral nerve tumors originating from Schwann cells. These tumors can occur spontaneously or be associated with familial conditions, such as neurofibromatosis type 2. Although schwannomas have various forms, they are classified as World Health Organization grade I tumors and rarely undergo malignant transformation.^[1]^ Most schwannomas are isolated, sporadic lesions, and many have mutations on chromosome 22.^[2]^ Schwannomas primarily occur in the head, neck, limbs, and trunk,^[3]^ while those arising in the mesocolon are rare.^[4]^ A literature search reveals that few such cases have been reported.

## 2. Case report

**Figure 1.**
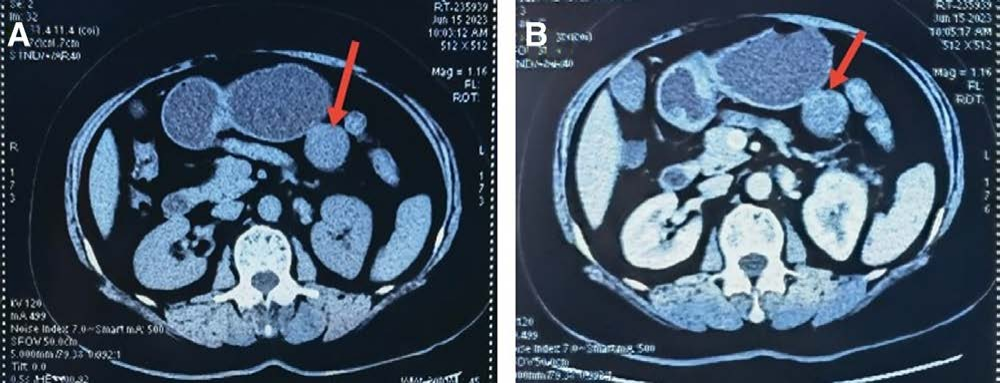
(A) A rounded, slightly hypodense mass was observed in the left abdominal cavity, with a CT value of about 22 HU. The upper edge of the mass was indistinctly demarcated from the greater curvature of the upper part of the gastric body. (B) A shadow of a less homogeneous mass was noted on the left side of the abdominal cavity, predominantly slightly hypodense with a well-defined border. The mass measured approximately 4.0 × 3.8 × 4.5 cm. The enhancement was less than homogeneous, with delayed enhancement visible in local areas. A blood vessel supplying the mass was identified, originating from the inferior mesenteric artery. The lesion was located close to, and was not clearly separated from, the greater curvature of the stomach.

A 59-year-old female patient presented to a local hospital with a 1-month history of acid reflux discomfort accompanied by dizziness. She was in good health with a body mass index of 26.56 and had no family history of neurofibromatosis. She had no history of smoking, drug use, or alcohol addiction. An abdominal computed tomography (CT) scan at the local hospital revealed an isolated abdominal mass (Fig. 1A). Physical examination of the abdomen did not reveal any palpable masses.

An enhanced CT scan of the abdomen revealed a mass of uneven density in the left side of the abdominal cavity, exhibiting heterogeneous and delayed enhancement. A blood vessel originating from the inferior mesenteric artery was observed, and the lesion was poorly demarcated from the greater curvature of the stomach (Fig. 1B). Based on these findings, the imaging diagnosis was a soft tissue lesion adjacent to the greater curvature of the stomach, considered to originate from mesenchymal tissue and suspected to be a stromal tumor.

To further clarify the diagnosis, the patient underwent gastroscopy, which did not reveal any obvious abnormalities. Comprehensive serological examinations, including ferritin (FER), carcinoembryonic antigen (CEA), alpha-fetoprotein (AFP), carbohydrate antigen 19-9 (CA19-9), cancer antigen 125 (CA125), and cancer antigen 15-3 (CA15-3), were all within normal limits. Based on this information, the hospital suspected that the mass was a gastric stromal tumor.

**Figure 2.**
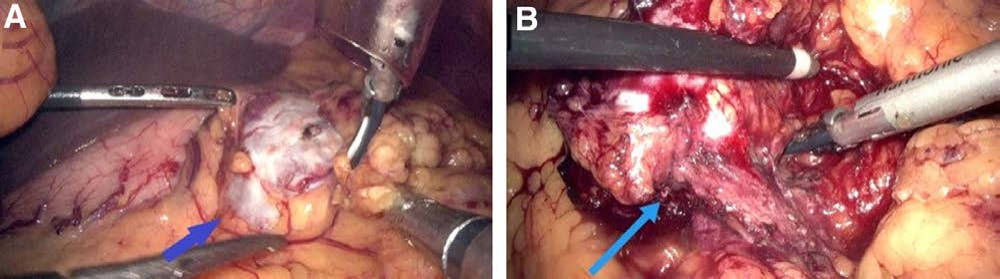
(A) Laparoscopic exploration revealed an abdominal mass. (B) Further exploration indicated that the mass is closely associated with the transverse colon.

Seeking further diagnosis and treatment, the patient presented to our hospital with the main complaint of “acid reflux for 1 month.” On June 26, 2023, she underwent laparoscopic surgery. During laparoscopy, the greater omentum was opened for exploration, revealing a mass measuring approximately 5 × 4 cm (Fig. 2A) that did not adhere to the fundus of the stomach, thus ruling out a gastric origin. Further exploration showed that the mass was closely related to the transverse mesocolon, and upon dissection, the remaining part of the tumor was found to be tightly adherent to the transverse colon (Fig. 2B). Intraoperatively, the mass was suspected to have originated from the transverse colon. The surgeon used a linear cutting stapler to resect the mass along with the adherent portion of the transverse colon. The procedure lasted 116 minutes without any postoperative complications. The patient recovered uneventfully and was discharged 9 days after surgery.

**Figure 3.**
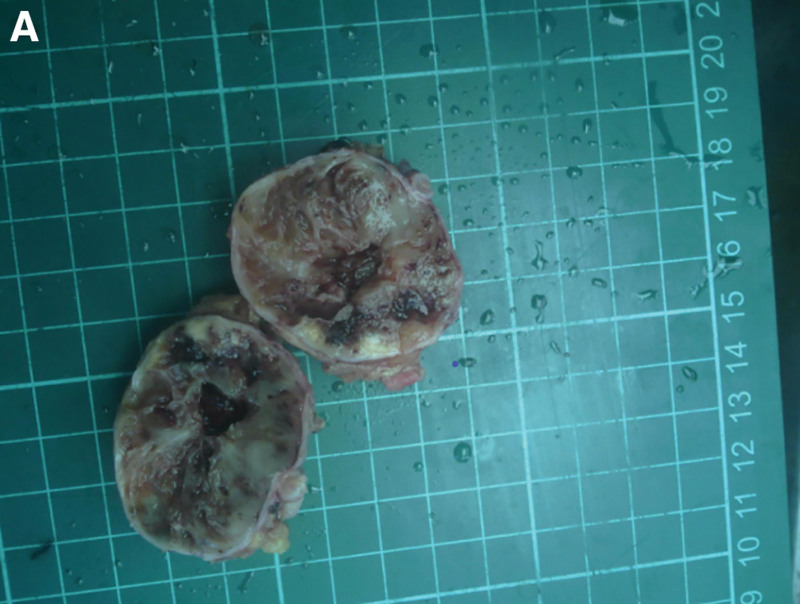
A nodular mass, measuring 4.5 × 3.5 × 3.5 cm, was observed. The mass was cystic-solid in consistency, featuring a cystic cavity measuring 2.5 cm in diameter. The solid area was grayish-white and grayish-yellow, soft, and locally adherent to part of the bowel wall.

**Figure 4.**
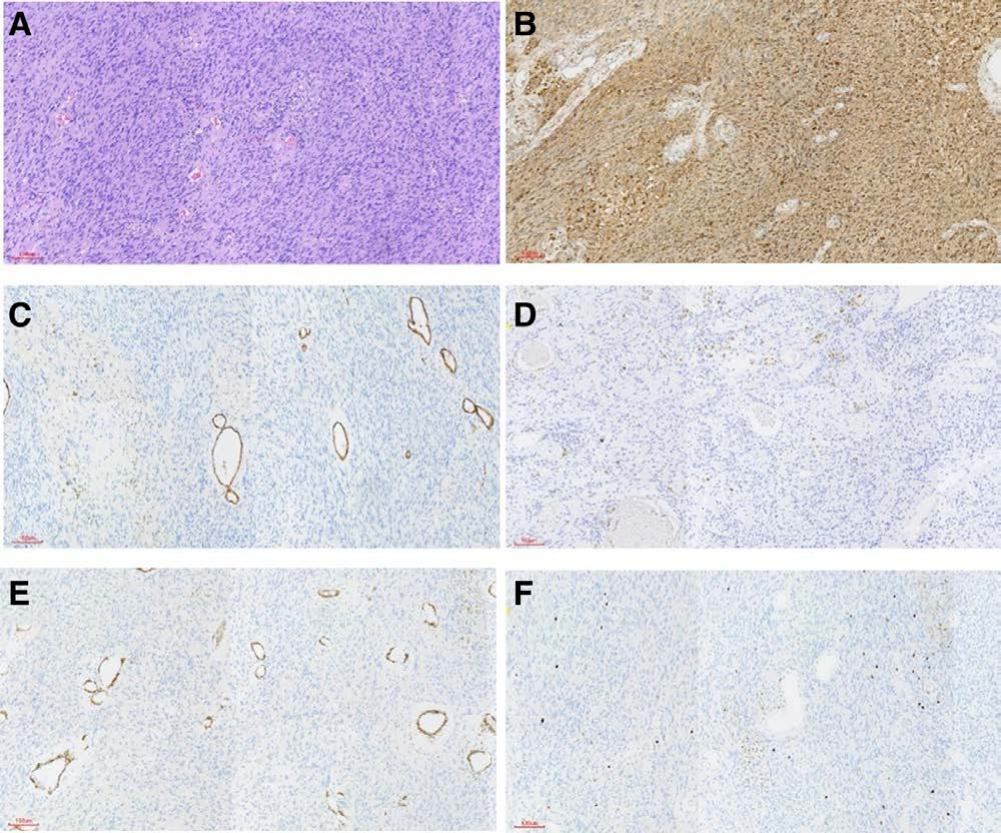
(A) A spindle cell tumor measuring 4.5 × 3.5 × 3.5 cm, exhibiting cystic degeneration, hemorrhage, and ferrous hemosiderin deposition. (B) S-100 protein was diffusely and strongly positive in the tumor cells. (C) CD34 was negative in the tumor cells, although normal vascular structures were positively stained. (D) CD-117 was negative in the tumor cells. (E) Smooth muscle actin (SMA) was negative in the tumor cells. (F) Ki-67 nuclear proliferation factor showed positive immunoexpression in <3% of the areas studied.

Gross examination of the resected mass showed it was cystic and solid (Fig. 3A). Routine postoperative pathological examination revealed a spindle cell tumor located outside the serosal surface of the intestinal wall, measuring 4.5 × 3.5 × 3.5 cm, with cystic changes, hemorrhage, hemosiderin deposition, and chronic inflammation of the overlying intestinal mucosa (Fig. 4A). Cystic changes and hemorrhage may represent secondary degenerative changes in schwannomas.^[5]^

Pathological findings refuted the diagnosis that the mass originated from the transverse colon because nerve sheath tumors located within the intestines tend to be concentrated in the intrinsic muscular layer.^[6,7]^ However, the mass did not invade the serosal layer of the transverse colon and was determined to originate from outside the transverse colon.

To further clarify the nature of the mass, immunohistochemical staining was performed, showing S-100 positive, CD34 positive (vascular), CD117 negative, SMA negative, and Ki-67 index of 3% (Fig. 4B–F). Based on all the diagnostic and therapeutic procedures, our team finally determined that the mass was a schwannoma of the transverse mesocolon.

The patient is now recovering well from the operation, and there was no sign of tumor recurrence at the 1-year postoperative follow-up.

## 3. Discussion

Schwannomas are peripheral nerve tumors originating from Schwann cells. These tumors can occur spontaneously or be associated with familial conditions such as neurofibromatosis type 2. Although they have various forms, schwannomas are classified as World Health Organization grade I tumors and rarely undergo malignant transformation.^[1]^ They primarily occur in the head, neck, limbs, and trunk,^[3]^ while mesocolic schwannomas are rare.^[4]^ Few cases have been reported in the literature.

In terms of clinical manifestations, schwannomas in the abdomen may produce symptoms due to compression of surrounding organs. These symptoms are nonspecific, and some patients may have no obvious clinical signs. In some cases, an isolated abdominal mass is discovered incidentally during examinations.^[8,9]^ Other patients may present with gastrointestinal symptoms, such as abdominal pain or acid reflux, and an intra-abdominal mass may be detected during a physical examination. When the tumor reaches a certain size, some patients may seek medical attention after noticing a palpable abdominal mass.^[4]^ Additionally, certain schwannomas may cause gastrointestinal obstruction due to pressure on the digestive tract.

For patients suspected of having gastrointestinal tumors, biopsies are often obtained via endoscopy, alongside enhanced CT scans and serological tests, to observe the tumor’s features and determine its nature. On CT, schwannomas often appear as round or oval masses with clear, low-density borders, and may exhibit cystic and hemorrhagic changes. Enhanced CT may show various degrees of uniform or uneven enhancement, with cystic and hemorrhagic changes contributing to the uneven enhancement.^[10]^ Since most abdominal schwannomas are located beneath the mucous membrane layer or in the submucosal space,^[6,7,11]^ obtaining biopsy specimens can be difficult, leading to challenges in making an accurate preoperative diagnosis.^[11]^ Final characterization usually requires pathological examination of postoperative specimens.

The case described in this report must be differentiated from colonic schwannomas. In a study of 20 patients with colonic and rectal schwannomas, Dr Markku Miettinen found that colonic schwannomas are more commonly found in the cecum, growing toward the intestinal lumen. These tumors can cause symptoms such as mucosal ulceration, bleeding, colonic obstruction, and abdominal pain. However, some patients may remain asymptomatic, with abdominal masses being discovered incidentally.^[6]^ In this case, colonoscopy did not reveal any submucosal bulge in the colon, and the patient experienced only acid reflux without other abdominal symptoms. The mass’s tight adherence to the transverse colon during surgery led to the initial misinterpretation of the tumor’s origin.

To differentiate schwannomas from gastrointestinal stromal tumors (GISTs), immunohistochemical staining is helpful. Schwannomas tend to express S-100 but not CD117, and only rarely express CD34. In contrast, GISTs typically express CD34 and CD117 but not S-100.^[6,11,12]^ The S-100 protein, a group of low-molecular-weight calcium-binding proteins, is highly conserved in vertebrates and predominantly found in human Schwann cells, Schwann-associated cells, satellite glial cells, and supporting cells in peripheral and paranodal ganglia. However, it is also present in other non-neural cell types and can be found in certain tumors derived from these cells.^[13,14]^

The mitotic index (Ki-67) is a useful indicator of a tumor’s benign or malignant potential.^[15]^ Benign schwannomas typically have low Ki-67 expression levels (0–6%, median 4%), while malignant schwannomas tend to have higher levels (12–45%, median 25%).^[16]^ A Ki-67 index >20% is highly predictive of malignant peripheral schwannomas.^[17]^ For patients with benign schwannomas, surgical excision can achieve excellent outcomes with a good prognosis.^[4,18]^ In contrast, malignant schwannomas require multimodal treatment, primarily based on surgery.^[19]^

Surgical resection is an effective treatment for abdominal schwannomas, with different surgical approaches available depending on the tumor’s location. Isolated schwannomas in the abdominal cavity can be treated with laparoscopic resection. Compared to traditional open surgery, laparoscopic procedures result in shorter hospital stays, faster recovery, and lower risks of tumor recurrence.^[20,21]^ Schwannomas located in the submucosal or intrinsic basal layers of the stomach and colon, growing into the lumen, can be treated with endoscopic resection.^[8,18]^

Finally, Table 1 presents a summary of the literature related to mesocolic schwannomas.

**Table 1:** Summary of selected mesenteric schwannoma.

Author	Publication year	Age (yr)	Sex	Clinical manifestation	Treatment	Tumour size (cm)	Radiographic examination	Tumor site	Follow-up (mo)	Any recurrence during follow-up
This example	–	59	Female	Acid reflux	Laparoscopic surgery	4.5 × 3.5 × 3.5	Computed tomography (CT), gastroscopy, colonoscopy	Mesentery	12	None
Ying-Sheng Wu et al.^[22]^	2018	58	Female	Asymptomatic	Surgeries	10.0 × 9.0 × 9.0	Ultrasound, CT, magnetic resonance imaging (MRI)	Mesentery	43	None
Shigeki Minami et al.^[4]^	2005	54	Female	Abdominal mass	Surgeries	8.0 × 7.0 × 4.8	CT, MRI	Jejunal mesentery	5	None
Bulent Kilicoglu et al.^[23]^	2006	56	Male	Nausea, vomiting, acute abdominal pain, constipation	Surgeries	22 × 19 × 4	Abdominal plain film	Mesentery of the small bowel	11	None
R. Murakami et al.^[24]^	1998	48	Male	Asymptomatic	Surgeries	4.5 × 4.0 × 4.0	CT, MRI, ultrasound	Mesentery	24	None
Inés Cañas García et al.^[9]^	2020	58	?	Stomach pain	Surgeries	8 × 6	CT, MRI	Mesentery	?	?
Alberto Abreu da Silva et al.^[25]^	2023	86	Male	Bloating and abdominal pain loss of appetite and vomiting	Surgeries	3.5 × 4 × 3.6	CT	Mesentery	24	None
S. Hagjer et al.^[26]^	2014	45	Female	Abdominal mass	Surgeries	15 × 14 × 13.5	Ultrasound	Mesentery of the small bowel	6	None
A. Tepox Padrón et al.^[27]^	2017	38	Female	Abdominal mass	Surgeries	11.3 × 8.4 × 4.1	MRI	Mesentery	24	None
S. Swaminathan et al.^[28]^	2019	32	Male	Abdominal pain	Surgeries	3.5 × 4.2 × 3.7	Ultrasound, CT	Mesentery of the small bowel	?	None

CT = computed tomography, MRI = magnetic resonance imaging.

## 4. Conclusion

In conclusion, transverse mesocolon schwannomas are rare and often present with nonspecific manifestations. In such cases, schwannomas closely adherent to surrounding normal tissues need to be assessed with the help of preoperative examinations, intraoperative observations, and postoperative specimen analysis to avoid misdiagnosis. Immunohistochemical staining is an important method to distinguish schwannomas from other gastrointestinal mesenchymal tumors, with markers like S-100 protein, CD34, and CD117 being particularly specific. Schwannomas are mostly benign tumors, and the Ki-67 index is one of the indicators used to assess the tumor’s benign or malignant potential. Benign schwannomas can be treated in different ways according to their growth patterns and locations, and the prognosis after surgical treatment is generally good.

## Author contributions

**Data curation:** Qihang Sun, Qingshun Zhu, Xuren Lu, Guangxu Zhu, Jie Zhang.

**Investigation:** Xuren Lu.

**Project administration:** Wei Lang, Jianjun Qu Doctor.

**Writing – original draft:** Qihang Sun.
